# Overexpression of Dimethylarginine Dimethylaminohydrolase 1 Attenuates Airway Inflammation in a Mouse Model of Asthma

**DOI:** 10.1371/journal.pone.0085148

**Published:** 2014-01-10

**Authors:** Kayla G. Kinker, Aaron M. Gibson, Stacey A. Bass, Brandy P. Day, Jingyuan Deng, Mario Medvedovic, Julio A. Landero Figueroa, Gurjit K. Khurana Hershey, Weiguo Chen

**Affiliations:** 1 Division of Asthma Research, Cincinnati Children's Hospital Medical Center, Cincinnati, Ohio, United States of America; 2 Department of Environmental Health, University of Cincinnati College of Medicine, Cincinnati, Ohio, United States of America; 3 Department of Chemistry, University of Cincinnati, Cincinnati, Ohio, United States of America; 4 Division of Allergy and Immunology, Cincinnati Children's Hospital Medical Center, Cincinnati, Ohio, United States of America; Harvard Medical School, United States of America

## Abstract

Levels of asymmetric dimethylarginine (ADMA), an endogenous inhibitor of nitric oxide synthase, are increased in lung, sputum, exhaled breath condensate and plasma samples from asthma patients. ADMA is metabolized primarily by dimethylarginine dimethylaminohydrolase 1 (DDAH1) and DDAH2. We determined the effect of DDAH1 overexpression on development of allergic inflammation in a mouse model of asthma. The expression of DDAH1 and DDAH2 in mouse lungs was determined by RT-quantitative PCR (qPCR). ADMA levels in bronchoalveolar lavage fluid (BALF) and serum samples were determined by mass spectrometry. Wild type and DDAH1-transgenic mice were intratracheally challenged with PBS or house dust mite (HDM). Airway inflammation was assessed by bronchoalveolar lavage (BAL) total and differential cell counts. The levels of IgE and IgG1 in BALF and serum samples were determined by ELISA. Gene expression in lungs was determined by RNA-Seq and RT-qPCR. Our data showed that the expression of DDAH1 and DDAH2 was decreased in the lungs of mice following HDM exposure, which correlated with increased ADMA levels in BALF and serum. Transgenic overexpression of DDAH1 resulted in decreased BAL total cell and eosinophil numbers following HDM exposure. Total IgE levels in BALF and serum were decreased in HDM-exposed DDAH1-transgenic mice compared to HDM-exposed wild type mice. RNA-Seq results showed downregulation of genes in the inducible nitric oxide synthase (iNOS) signaling pathway in PBS-treated DDAH1-transgenic mice versus PBS-treated wild type mice and downregulation of genes in IL-13/FOXA2 signaling pathway in HDM-treated DDAH1-transgenic mice versus HDM-treated wild type mice. Our findings suggest that decreased expression of DDAH1 and DDAH2 in the lungs may contribute to allergic asthma and overexpression of DDAH1 attenuates allergen-induced airway inflammation through modulation of Th2 responses.

## Introduction

Asthma is a chronic inflammatory lung disease characterized by airway hyperresponsiveness (AHR), airway inflammation, excess mucus production and pulmonary remodeling [1]. Arginine metabolism has been found to play a critical role in the pathogenesis of allergic asthma [1], [2] and mounting evidence suggests that nitric oxide (NO) bioavailability plays an important role in the development of allergic inflammation [3], [4]. Arginine is metabolized by arginase yielding urea and L-ornithine that is further metabolized to polyamines and prolines, which can modulate cell proliferation and collagen production [4]. Arginine is also metabolized by nitric oxide synthases (NOS) including neuronal NOS (nNOS), inducible NOS (iNOS) and endothelial NOS (eNOS) [5]. eNOS is expressed in bronchial epithelium and type II alveolar epithelium, nNOS is expressed in airway nervous tissue, and iNOS is expressed in type II alveolar epithelium, lung fibroblasts, airway and vascular smooth muscle cells. The constitutive NO produced by nNOS and eNOS is important for smooth muscle relaxation, bronchodilation and determination of vascular tone and blood pressure while the inducible NO produced by iNOS has pro-inflammatory effects [5].

Asymmetric dimethylarginine (ADMA) is a by-product released from proteolysis of methylated proteins and ADMA competitively inhibits all three NOS by displacing L-arginine from NOS [6]. ADMA also competes with arginine for cellular uptake by cationic amino-acid transporters, affecting the cellular ADMA/arginine ratio [7], [8]. Analysis of methylarginine metabolism in the cardiovascular system showed that the lung is a major source of ADMA [9]. ADMA has been shown to have profound effects on multiple tissues. Microarray studies showed that pathophysiological concentrations of ADMA elicit significant changes in the gene expression in coronary artery endothelial cells [10]. Treatment of primary mouse lung fibroblasts with ADMA induced arginase activity and collagen production [11]. ADMA infusion resulted in increased lung resistance and decreased compliance in response to methacholine in mice, which was associated with significantly increased pulmonary collagen deposition [11]. ADMA potentiates ovalbumin-induced airway inflammation in a mouse model of asthma [12]. In humans, plasma ADMA levels are increased in severe asthma patients compared to nonsevere asthma patients and control subjects [3]. Other studies showed that ADMA levels are increased in sputum and exhaled breath condensate from asthma patients [13]–[15]. A more recent study showed that lower L-arginine/ADMA ratios are associated with reduced lung function and increased respiratory symptom frequency in subjects with late-onset asthma [16].

DDAH activity is a key determinant of intracellular ADMA concentration [17]. Ninety percent of ADMA is metabolized by DDAH and the rest is excreted through the kidneys. DDAH metabolizes ADMA to generate citrulline and dimethylamine. There are two isoforms of DDAH in human, mouse, rat and other species, DDAH1 and DDAH2 [18]–[20]. Immunostaining of human lung tissues showed expression of DDAH1 and DDAH2 in both alveolar and bronchiolar epithelium [21]. One study showed that homozygous DDAH1-deficient mice die before birth while the heterozygous DDAH1-deficient mice have a 20% increased level of ADMA and develop severe endothelial dysfunction [22]. Anther study showed that DDAH1-deficient mice are viable and have significantly increased ADMA levels [23]. ADMA levels are decreased in DDAH1-transgenic mice by 50% compared to wild type mice. Further, DDAH1-transgenic mice display a significant increase in NOS activity and a decreased risk of endothelial dysfunction compared to the wild type mice [24]–[26]. DDAH1 is the major enzyme responsible for metabolizing ADMA whereas DDAH2 has no detectable role in degrading ADMA *in vivo* [23]. In this paper, we determined the role of DDAH1 in the development of AHR and airway inflammation.

## Materials and Methods

### Mice

Mice were maintained and handled under Institutional Animal Care and Use Committee-approved procedures (Cincinnati Children's Hospital Medical Center, Protocol Number: 2D10082) and the Guide for the Care and Use of Laboratory Animals (Institute of Laboratory Animal Resources, National Research Council). C57BL/6 wild type and DDAH1-transgenic mice were purchased from Jackson Laboratory (Bar Harbor, ME).

### Immunization

C57BL/6 wild type and DDAH1-transgenic mice were immunized with intratracheal instillation of 100 µg of house dust mite (HDM) (*Dermatophagoides pteronyssinus*) extract (Greer Laboratories, Lenoir, NC) in 50 µl of PBS or 50 µl of PBS alone 3 times per week for 3 weeks as previously described [27], [28].

### Analysis of AHR and airway inflammation

Twenty four hours after the last treatment, AHR in response to methacholine (0, 50, 100 and 200 mg/ml) was measured by flexiVent (SCIREQ, Montreal, Canada) as previously described [29]. After AHR measurement, blood, bronchoalveolar lavage (BAL) samples and lung tissues were harvested as previously described [30]. The BAL total and differential cell counting as well as histological staining of lung sections by hematoxylin and eosin (H&E) or periodic acid Schiff (PAS) (Thermo Fisher Scientific, Waltham, MA) were performed as described by the manufacturer or described previously [30]. ELISAs for total IgE and IgG1 or HDM-specific IgE and IgG1 in BAL fluid (BALF) and serum were done as previously described [31].

### RNA-Seq and Bioinformatics Analysis

Total RNA was isolated from mouse lungs using Trizol reagent (Invitrogen, Carlsbad, CA), digested with RNase-free DNase and purified using an RNeasy MinElute kit (QIAGEN, Valencia, CA). Equal amounts of RNA were pooled from each mouse lung in an experimental group (n = 4 per group) and analyzed in duplicate. RNA-Seq was performed by the Genomics Sequencing Core in the University of Cincinnati. The RNA-Seq library was constructed using a PrepX SPIA RNA-Seq kit (IntegenX, Pleasanton, CA) and Apollo 324 NGS Library Prep System (IntegenX). 10 ng of total RNA was converted into cDNA suitable for mRNA sequencing. The cDNA was then sheared by Covaris S2 (Covaris, Woburn, MA) under the conditions recommended by IntegenX, followed by Bioanalyzer assay of the size distribution with Agilent High Sensitivity DNA kit (Agilent, Santa Clara, CA). The properly sheared cDNA fragments were purified by Agencourt AMPure XP magnetic beads (Beckman Coulter, Brea CA). Using the IntegenX PrepX ILM DNA library kit for Illumina and Apollo 324 NGS Library Prep System, 500 ng of purified cDNA fragments were then put through end repair, addition of a single ‘A’ base and ligation of adapters, and indexed individually. The products were purified and enriched by PCR to create the final cDNA library targeting mRNAs. The size of the generated library was validated by Bioanalyzer and the library was quantified using the Kapa Library Quantification kit (Kapa Biosystems, Woburn, MA). Six individually indexed cDNA libraries were equal amount pooled for clustering in cBot system (Illumina, San Diego, CA). Libraries at the concentration of 6.5 pM were clustered onto a flow cell using Illumina's TruSeq SR Cluster Kit v3, and sequenced for 50 cycles using the TruSeq SBS kit on Illumina HiSeq system.

Sequence reads were aligned to the reference genome (mm10) using TopHat aligner [32]. The counts of reads aligning to each gene's coding region were summarized using ShortRead and associated Bioconductor packages for manipulating and analysis of next-generation sequencing data and custom-written R programs [33], [34]. Statistical analysis to identify differentially expressed genes for each comparison was performed using the negative-binomial model of read counts as implemented in the DESeq Biocondoctor package [35]. P-values were adjusted for multiple comparisons based on false discovery rates (FDR) [36]. Differential expressions with adjusted p-values of <0.05 were considered statistically significant.

Functional enrichment analysis was performed using the logistic regression based LRpath methodology [37]. The gene lists used in the functional enrichment analysis were from genes associated with Gene Ontology terms and KEGG pathways. The statistical significance of gene list enrichment was determined by the False Discovery Rate cut-off of 0.1. Genes that were both members of at least one statistically significant gene list and had differential expression p-values of <0.01 were considered to be differentially expressed for the purpose of network analysis. Ingenuity Pathways Analysis (IPA) (Ingenuity Systems, Mountain View, CA) was used to identify integrated and interconnected biological networks and upstream targets for the differentially expressed genes between two groups (with adjusted p-values of <0.05). RNA-Seq data have been deposited with the NCBI Gene Expression Omnibus (http://www.ncbi.nlm.nih.gov/geo) under accession number GSE49047.

### RT-PCR

Total RNA was isolated from lung tissues using Trizol reagent (Invitrogen), digested with RNase-free DNase and purified using an RNeasy MinElute kit (QIAGEN). Reverse transcription was done using Oligo-dT First-Strand cDNA Synthesis Kit (GE Healthcare, Piscataway, NJ). Quantitative PCR (qPCR) was done using the SYBR Green Master Kit and LightCycler® 480 instrument (Roche Diagnostics, Indianapolis, IN). All primers used are listed in Table S1.

### Mass Spectrometry (MS)

MS was performed by the Analytical & Mass Spectrometry of Small Molecules Core at the University of Cincinnati. The procedure to quantify ADMA was adapted from Schwedhelm *et al* [38]. In brief, BALF and serum samples were filtrated through 0.45 µm filters to remove particles and 200 µl aliquots were then ultra-filtrated through 3 kDa MWCO filters to remove proteins. The samples were derivatized by adding 200 µl of 2 M HCl in 1-butanol for 20 min at 65°C. After evaporation, samples were reconstituted in 200 µl of distilled deionized water and analyzed by nano liquid chromatography chip electrospray ionization ion trap MS (nanoLC-Chip ESI-IT-MS). For nanoLC-Chip ESI-MS/MS analysis, the derivatized ADMA was separated in a microfluidic reverse phase chip column and detected by electrospray ionization with ion trap MS/MS detection. The tandem system consisted of an Agilent 1200 HPLC (Agilent Technologies, Santa Clara, CA), equipped with a capillary and nano pump, used for loading and flushing the on-chip nano column, a chip cube interface that contains the nano-chip column Zorbax SB C-18, 150×0.75 mm (Agilent Technologies), and an Agilent 6300 ion trap XCT system (Agilent Technologies). The mobile phase A consisted of 0.1% formic acid in water while B consisted of 0.1% formic acid in a 7:1 acetonitrile:water solution. A linear gradient from 2% to 25% B was carried out in 10 min and the column was then cleaned with 100% B for 5 min and then regenerated at original conditions for 10 min before the next injection. The flow rate was 0.3 µl per min and the outlet of the column communicates directly with the nano electrospray needle. The analysis was carried out in the MRM mode by following the transition m/z 259.3→214. ADMA standards (Sigma-Aldrich, St. Louis, MO) were derivatized in the same way and quantification was carried out by the external calibration method.

### Statistical analysis

All values are expressed as mean ± SD. The data were analyzed with a 2-tailed unpaired student's t-test with Welch's correction or 1-way ANOVA with Newman-Keuls' post test using Prism 5.0c for Mac OS X from GraphPad Software (San Diego, CA). A p-value of <0.05 was considered statistically significant.

## Results

### Expression of DDAH1 and DDAH2 is decreased in the lungs and ADMA levels are increased in BALF and serum from HDM-treated mice

**Figure 1:**
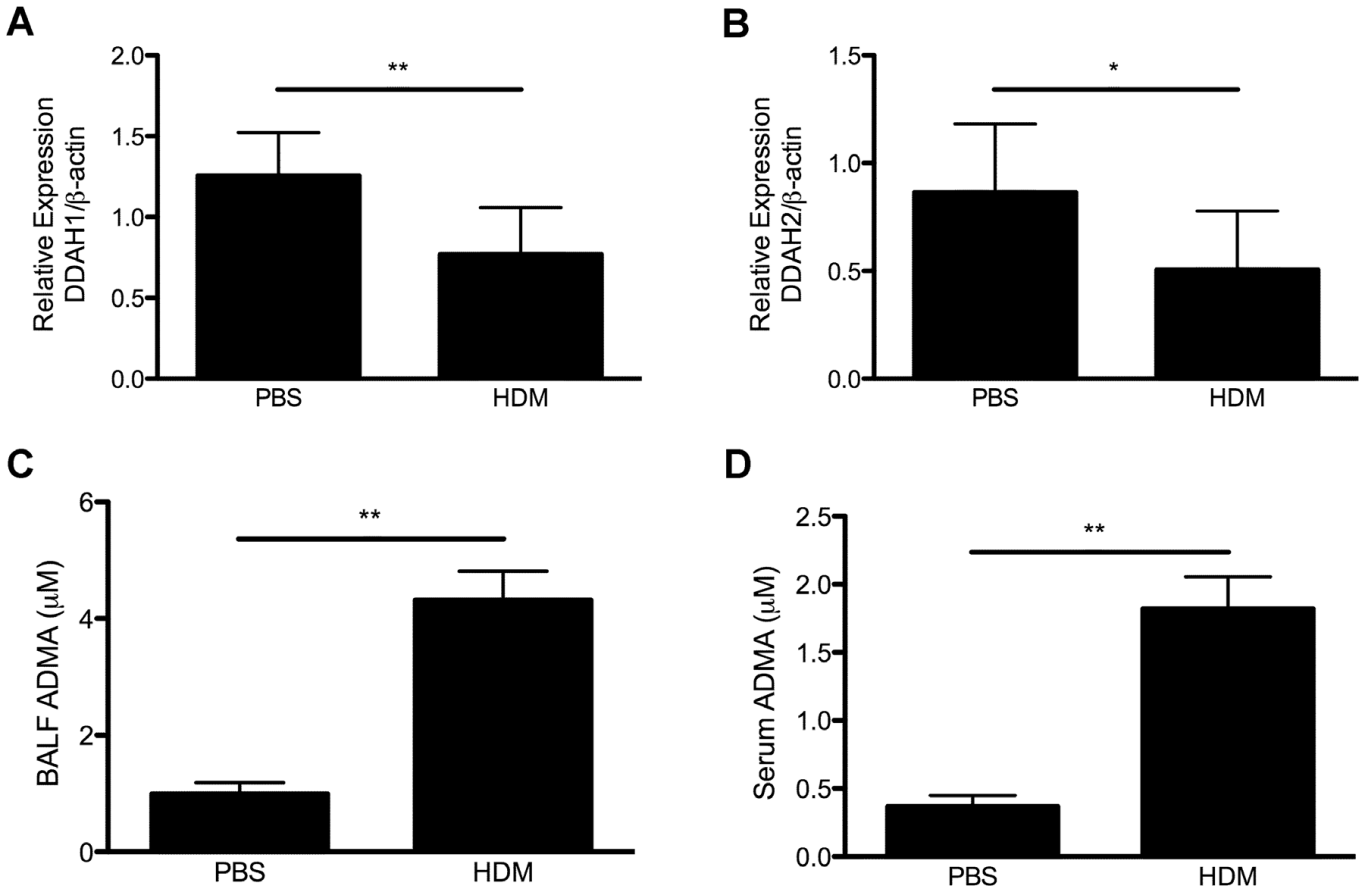
Expression of DDAH1 and DDAH2 in lungs and ADMA levels in BALF and serum samples from PBS or HDM-treated C57BL/6 mice. The mice were treated with PBS or HDM by 3 intratracheal challenges per week for 3 weeks. (A–B) DDAH1 and DDAH2 expression in lungs (n = 7–9). (C–D) ADMA levels in BALF and serum samples (n = 3). Data are shown as mean±SD. *, p<0.05; **, p<0.01.

Since ADMA levels are increased in allergic asthma and DDAH is responsible for the majority of ADMA metabolism, we hypothesized that the increased levels of ADMA are due to decreased expression of DDAH following allergen exposure. We determined the expression of DDAH1 and DDAH2 in the lungs in a mouse model of asthma. C57BL/6 mice were challenged by intratracheal instillation of 100 µg HDM, 3 times per week for 3 weeks. This is a more clinically relevant protocol of chronic allergen exposure with an increased dosage of HDM for the C57BL/6 mouse strain. As shown in Fig. 1A and 1B, HDM exposure resulted in decreased expression of DDAH1 and DDAH2 in mouse lungs, which correlated with increased levels of ADMA in BALF and serum (Fig. 1C and 1D).

### Overexpression of DDAH1 attenuates HDM-induced airway inflammation

**Figure 2:**
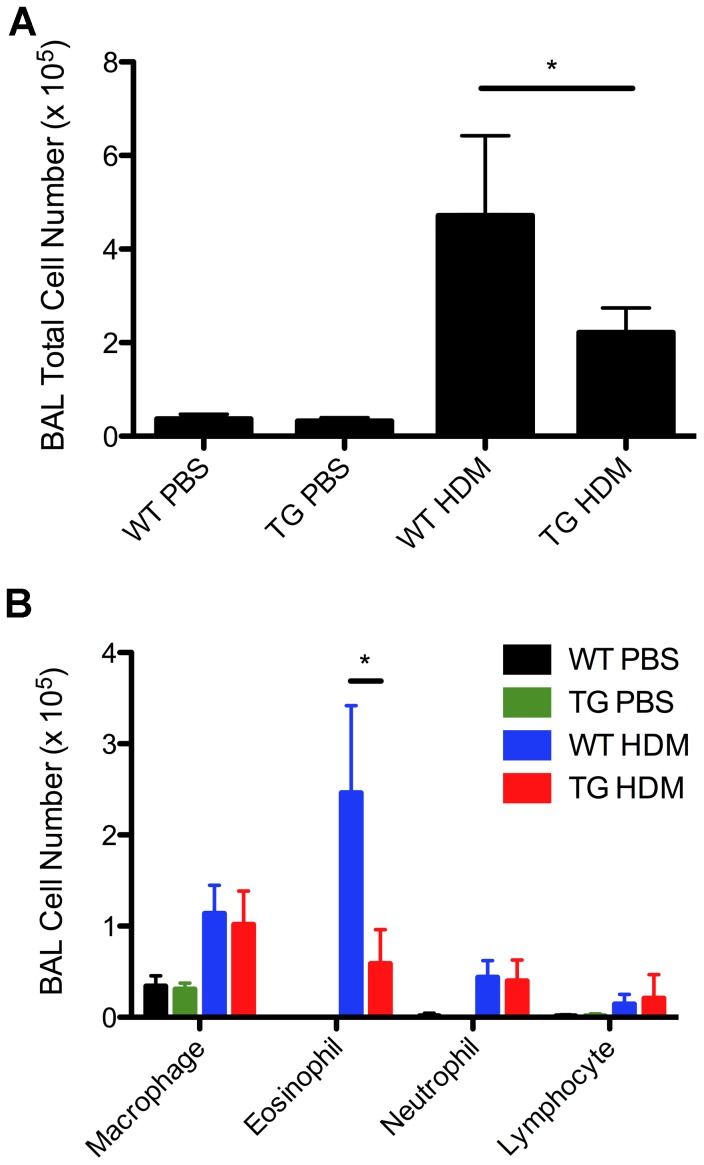
Airway inflammation in PBS or HDM-treated C57BL/6 wild type and DDAH1-transgenic mice. BAL total and differential cell counts in wild type and DDAH1-transgenic mice treated with PBS or HDM (3 intratracheal challenges per week for 3 weeks). WT: wide type; TG: DDAH1-transgenic. Data are shown as mean±SD (n = 5–9). *, p<0.05.

We next determined the effect of DDAH overexpression on airway inflammation and AHR in a HDM-induced asthma model using DDAH1-transgenic mice since DDAH1 is the major enzyme responsible for ADMA metabolism [23]. The mice were challenged by intratracheal instillation of 100 µg HDM 3 times per week for 3 weeks. Twenty-four hours after the last treatment AHR was measured by flexiVent and airway inflammation was assessed by BAL cell counts. BAL total cell and eosinophil numbers were decreased in HDM-treated DDAH1-transgenic mice compared to HDM-treated wild type mice (Fig. 2A-2B). No significant differences in inflammatory cell infiltration in airways determined by H&E staining or in mucus production determined by PAS staining of lung sections were observed between HDM-treated DDAH1-transgenic mice and HDM-treated wild type mice (data not shown). No significant difference in AHR was observed in HDM-treated DDAH1-transgenic mice compared to HDM-treated wild type mice (data not shown).

### IgE levels in BALF and serum are decreased in DDAH1-transgenic mice compared to wild type mice after HDM treatment

**Figure 3:**
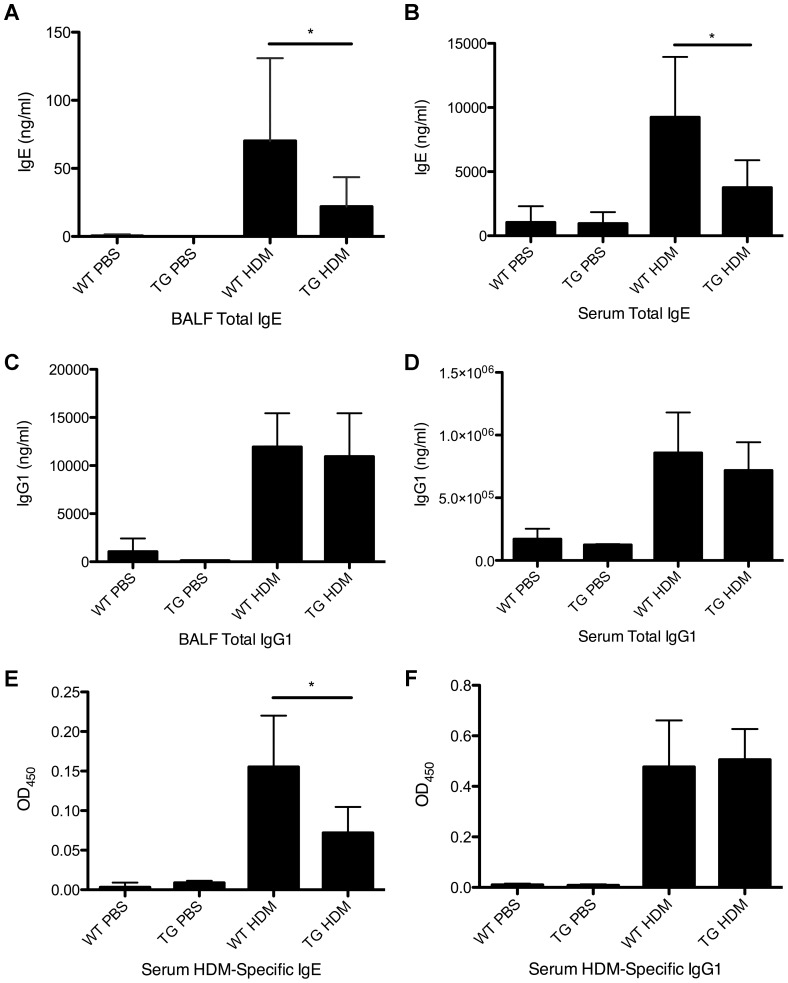
Levels of IgE and IgG1 in BALF and serum in PBS or HDM-treated C57BL/6 wild type and DDAH1-transgenic mice. (A) BALF total IgE. (B) Serum total IgE. (C) BALF total IgG1. (D) Serum total IgG1. (E) Serum HDM-specific IgE. (F) Serum HDM-specific IgG1. WT: wide type; TG: DDAH1-transgenic. Data are shown as mean±SD (n = 5–9). *, p<0.05.

We next tested whether allergen sensitization was affected by the overexpression of DDAH1. As shown in Fig. 3A and 3B, the total IgE levels were decreased in BALF and serum in HDM-treated DDAH1-transgenic mice compared to HDM-treated wild type mice but there was no difference in the levels of total IgG1 in BALF or serum (Fig. 3C and 3D). The level of HDM-specific IgE was decreased in serum from HDM-treated DDAH1-transgenic mice compared to HDM-treated wild type mice but the level of HDM-specific IgG1 in serum was unaffected (Fig. 3E and 3F).

### Gene expression profiles are significantly different in the lungs of wild type and DDAH1-transgenic mice

To elucidate the potential mechanisms underlying attenuated HDM-induced airway inflammation in DDAH1-transgenic mice, we determined gene expression profiles in the lungs from C57BL/6 wild type and DDAH1-transgenic mice treated with PBS or HDM (intratracheal instillation of 100 µg of HDM in 50 µl of PBS or 50 µl of PBS alone 3 times per week for 3 weeks) by RNA-Seq.

**Figure 4:**
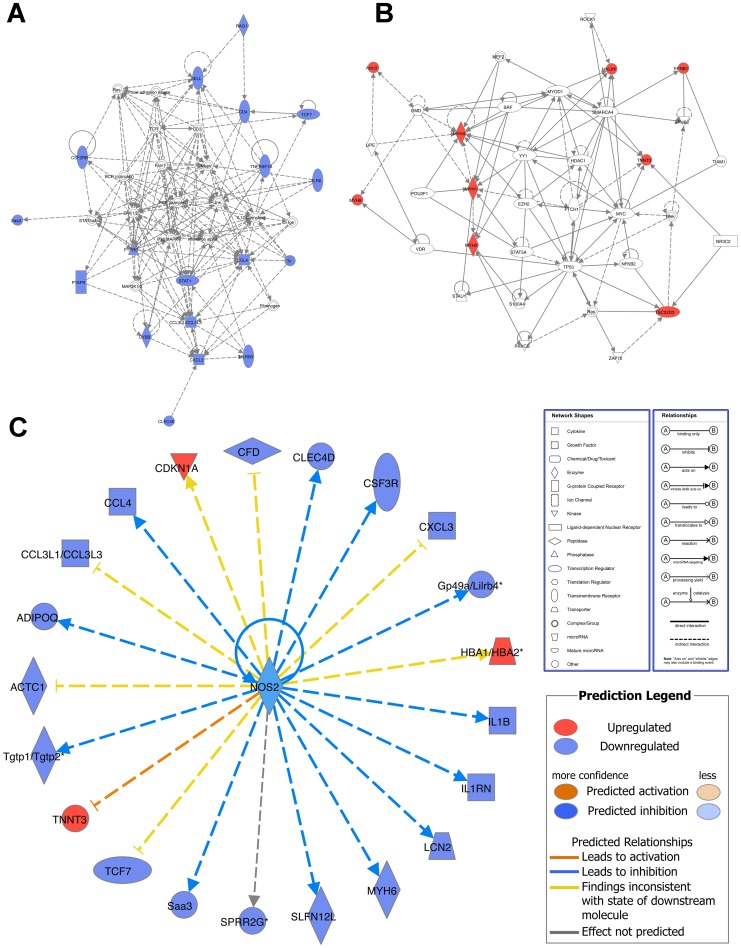
Network analysis of differentially expressed genes in the lungs of PBS-treated C57BL/6 wild type and PBS-treated DDAH1-transgenic mice. (A) Top network of the downregulated immune/defense response genes, cytokine/chemokine genes and transcriptional factor genes in PBS-treated DDAH1-transgenic mice. (B) Top network of the upregulated muscle/cell structure genes in PBS-treated DDAH1-transgenic mice. (C) Upstream target analysis of genes differentially expressed in PBS-treated wild type and DDAH1-transgenic mice. The genes with an adjusted p-value of <0.05 were included for analysis.

There were 354 genes differentially expressed in the lungs of PBS-treated wild type and PBS-treated DDAH1-transgenic mice (Table S2). The top downregulated genes (with an adjusted p-value of <0.05) are shown in Table 1 (grouped and sorted by fold change), including immune/defense response genes (Rag1, Irg1, Tlr6, etc.), cell structure/adhesion/migration genes (Sprr2a2, Sprr2a1, Adipoq, etc.), cytokine/chemokine genes (Ccl4, Ccl3, Cxcl2 and Il1b), and transcriptional factors (Tcf7 and Stat1). The top upregulated genes (with an adjusted p-value of <0.05) are shown in Table 2 (grouped and sorted by fold change), including muscle/cell structure genes (Myh2, Myh8, Myh4, etc.), ion homeostasis/metabolism genes (Atp2a1, Ckm, Sod1, etc.) and hemopoiesis genes (Hba-a2, Hba-a1, Beta-s, etc.). For the downregulated immune/defense response genes, cytokine/chemokine genes and transcriptional factor genes, IPA analysis showed that the top network was associated with inflammatory response, immunological disease, and respiratory disease (Fig. 4A). The top network for the upregulated muscle/cell structure genes was associated with organ morphology, skeletal and muscular system development and function, and cancer (Fig. 4B). The top upstream target for both downregulated and upregulated genes was iNOS (NOS2) (Fig. 4C).

**Table 1:** Genes Downregulated in the Lungs of PBS-treated DDAH1-transgenic Mice versus PBS-treated Wild Type Mice.

Symbol	Name	Fold Change
***Immune/Defense Response***		
Rag1	recombination activating gene 1	0.0020
Irg1	immunoresponsive gene 1	0.0042
Tlr6	toll-like receptor 6	0.0224
Saa3	serum amyloid A 3	0.0265
Clec4e	C-type lectin domain family 4, member e	0.0360
Ptafr	platelet-activating factor receptor	0.0656
Pram1	PML-RAR alpha-regulated adaptor molecule 1	0.1024
Clec4d	C-type lectin domain family 4, member d	0.1306
Cfd	complement factor D (adipsin)	0.1491
Cd4	CD4 antigen	0.2667
Tnfrsf1b	tumor necrosis factor receptor superfamily, member 1b	0.3327
Il1rn	interleukin 1 receptor antagonist	0.3365
Lilrb3	leukocyte immunoglobulin-like receptor, subfamily B (with TM and ITIM domains), member 3	0.3596
Sell	selectin, lymphocyte	0.3826
Acsl1	acyl-CoA synthetase long-chain family member 1	0.4620
Cybb	cytochrome b-245, beta polypeptide	0.4694
Lcn2	lipocalin 2	0.4804
Tgtp1	T-cell specific GTPase 1	0.4907
Lilrb4	leukocyte immunoglobulin-like receptor, subfamily B, member 4	0.4931
Tgtp2	T-cell specific GTPase 2	0.4986
Csf2rb	colony stimulating factor 2 receptor, beta, low-affinity (granulocyte-macrophage)	0.5378
Ptprc	protein tyrosine phosphatase, receptor type, C	0.6052
***Cell Structure/Adhesion/Migration***		
Sprr2a2	small proline-rich protein 2A2	0.0179
Sprr2a1	small proline-rich protein 2A1	0.0179
Adipoq	adiponectin, C1Q and collagen domain containing	0.0702
S100a9	S100 calcium binding protein A9 (calgranulin B)	0.0736
Pla2g7	phospholipase A2, group VII (platelet-activating factor acetylhydrolase, plasma)	0.1651
S100a8	S100 calcium binding protein A8 (calgranulin A)	0.2070
H2-M2	histocompatibility 2, M region locus 2	0.2210
Sema4d	sema domain, immunoglobulin domain (Ig), transmembrane domain (TM) and short cytoplasmic domain, (semaphorin) 4D	0.2948
Itgb2	integrin beta 2	0.4746
Myh6	myosin, heavy polypeptide 6, cardiac muscle, alpha	0.4752
Itgal	integrin alpha L	0.4913
Actc1	actin, alpha, cardiac muscle 1	0.5662
Csf3r	colony stimulating factor 3 receptor (granulocyte)	0.5942
***Cytokine/Chemokine***		
Ccl4	chemokine (C-C motif) ligand 4	0.0000
Ccl3	chemokine (C-C motif) ligand 3	0.0114
Cxcl2	chemokine (C-X-C motif) ligand 2	0.0236
Il1b	interleukin 1 beta	0.1342
***Transcriptional Factor***		
Tcf7	transcription factor 7, T-cell specific	0.4134
Stat1	signal transducer and activator of transcription 1	0.5833
***Other***		
Arpp21	cyclic AMP-regulated phosphoprotein, 21	0.0036
Gxylt2	glucoside xylosyltransferase 2	0.0112
1700071M16Rik	RIKEN cDNA 1700071M16 gene	0.0427
Trim30b	tripartite motif-containing 30B	0.0516
Cd177	CD177 antigen	0.0746
Niacr1	niacin receptor 1	0.0993
Mmp8	matrix metallopeptidase 8	0.1307
Slfn4	schlafen 4	0.1469
Acpp	acid phosphatase, prostate	0.2390
Steap4	STEAP family member 4	0.2412
F13a1	coagulation factor XIII, A1 subunit	0.2631
Gpnmb	glycoprotein (transmembrane) nmb	0.3398
Slpi	secretory leukocyte peptidase inhibitor	0.3547
Bpifa1	BPI fold containing family A, member 1	0.3796
Gp49a	glycoprotein 49 A	0.4032
Car3	carbonic anhydrase 3	0.4062
Gm1966	predicted gene 1966	0.5319
Ctss	cathepsin S	0.6001
Fth1	ferritin heavy chain 1	0.6449

NCBI Gene Expression Omnibus accession numbers GSE49047 (http://www.ncbi.nlm.nih.gov/geo/query/acc.cgi?acc=GSE49047).

**Table 2:** Genes Upregulated in the Lungs of PBS-treated DDAH1-transgenic Mice versus PBS-treated Wild Type Mice.

Symbol	Name	Fold Change
***Muscle/Cell Structure***		
Myh2	myosin, heavy polypeptide 2, skeletal muscle, adult	181.3505
Myh8	myosin, heavy polypeptide 8, skeletal muscle, perinatal	109.1566
Myh4	myosin, heavy polypeptide 4, skeletal muscle	82.8001
Myh1	myosin, heavy polypeptide 1, skeletal muscle, adult	65.6757
Tnnt3	troponin T3, skeletal, fast	44.0881
Actn3	actinin alpha 3	26.0940
Mylpf	myosin light chain, phosphorylatable, fast skeletal muscle	14.9051
Neb	nebulin	10.4391
***Ion Homeostasis/Metabolism***		
Atp2a1	ATPase, Ca++ transporting, cardiac muscle, fast twitch 1	45.0004
Ckm	creatine kinase, muscle	9.6517
Sod1	superoxide dismutase 1, soluble	1.8226
Errfi1	ERBB receptor feedback inhibitor 1	1.7924
Dpep1	dipeptidase 1 (renal)	1.6845
Sgms1	sphingomyelin synthase 1	1.6440
Glul	glutamate-ammonia ligase (glutamine synthetase)	1.4929
***Hemopoiesis***		
Hba-a2	hemoglobin alpha, adult chain 2	1.6691
Hba-a1	hemoglobin alpha, adult chain 1	1.6691
Beta-s	hemoglobin subunit beta-1-like	1.5436
Hbb-b1	hemoglobin, beta adult major chain	1.5242
Hbb-b2	hemoglobin, beta adult minor chain	1.5242
***MicroRNA***		
Mir5109	microRNA 5109	2.7839
***Other***		
Flrt2	fibronectin leucine rich transmembrane protein 2	11.5849
Gm13375	predicted gene 13375	8.1901
Gm3893	predicted gene 3893	5.6492
Rmrp	RNA component of mitochondrial RNAase P	4.8670
Angptl4	angiopoietin-like 4	2.7019
Efnb1	ephrin B1	2.3349
Zbtb16	zinc finger and BTB domain containing 16	2.2658
Cdkn1a	cyclin-dependent kinase inhibitor 1A (P21)	2.2030
Tsc22d3	TSC22 domain family, member 3	2.1869
6430548M08Rik	RIKEN cDNA 6430548M08 gene	2.1316
Krt7	keratin 7	2.0891
Bmp6	bone morphogenetic protein 6	1.8952
Gm10393	predicted gene 10393	1.8802
Lars2	leucyl-tRNA synthetase, mitochondrial	1.8365
Plxna2	plexin A2	1.8323
Eif3e	eukaryotic translation initiation factor 3, subunit E	1.7913
Cpm	carboxypeptidase M	1.7450
Rn45S	45S pre-ribosomal RNA	1.7382
Vps54	vacuolar protein sorting 54 (yeast)	1.5716
Dnaja1	DnaJ (Hsp40) homolog, subfamily A, member 1	1.5133
Tmbim6	transmembrane BAX inhibitor motif containing 6	1.4524
Sepp1	selenoprotein P, plasma, 1	1.3714
Sftpa1	surfactant associated protein A1	1.3527

NCBI Gene Expression Omnibus accession numbers GSE49047 (http://www.ncbi.nlm.nih.gov/geo/query/acc.cgi?acc=GSE49047).

**Figure 5:**
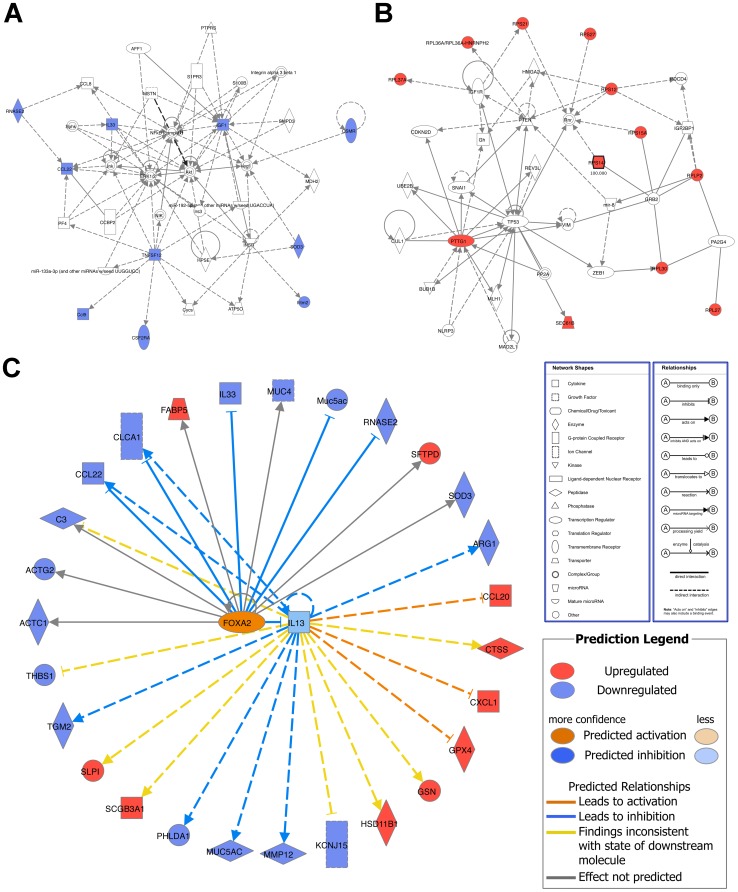
Network analysis of differentially expressed genes in the lungs of HDM-treated C57BL/6 wild type and HDM-treated DDAH1-transgenic mice. (A) Top network of the downregulated immune/defense response genes and cytokine/chemokine genes in HDM-treated DDAH1-transgenic mice. (B) Top network of the upregulated ribosomal complex genes and ribosomal pseudogenes in HDM-treated DDAH1-transgenic mice. (C) Upstream target analysis of genes differentially expressed in HDM-treated wild type and DDAH1-transgenic mice. The genes with an adjusted p-value of <0.05 were included for analysis.

There were 707 genes differentially expressed in the lungs of HDM-treated wild type and HDM-treated DDAH1-transgenic mice (Table S3). The top downregulated genes (with an adjusted p-value of <0.05) are shown in Table 3 (grouped and sorted by fold change), including metabolism/transport genes (Fbp1, Dhrs9, Arg1, etc.), immune/defense response genes (Ear11, Chia, Sod3, etc.), cytokine/chemokine genes (Ccl22, Tnfsf12, Ccl9 and Il33), extracellular matrix genes (Col6a2, Muc5ac, Muc4 and Col1a1) and microRNAs (Mir5109 and Mir5107). The top upregulated genes (with an adjusted p-value of <0.05) are shown in Table 4 (grouped and sorted by fold change), including ribosomal complex genes (Rps21, Rps12, Rpl30, etc.) and ribosomal pseudogenes (Gm11968, Gm12191, Gm13253, etc.). The top network for the downregulated immune/defense genes and cytokine/chemokine genes was associated with inflammatory response, cellular movement, and hematological system development and function (Fig. 5A). The top network for the upregulated ribosomal complex genes and ribosomal pseudogenes is associated with cellular development, cancer, and cellular assembly and organization (Fig. 5B). The top upstream targets for both downregulated and upregulated genes were IL-13 and FOXA2 (Fig. 5C).

**Table 3:** Genes Downregulated in the Lungs of HDM-treated DDAH1-transgenic Mice versus HDM-treated Wild Type Mice.

Symbol	Name	Fold Change
***Metabolism/Transport***		
Fbp1	fructose bisphosphatase 1	0.1302
Dhrs9	dehydrogenase/reductase (SDR family) member 9	0.1595
Arg1 *	arginase, liver	0.1808
Slc26a4	solute carrier family 26, member 4	0.2789
Cox15	COX15 homolog, cytochrome c oxidase assembly protein (yeast)	0.2889
Chi3l4 *	chitinase 3-like 4	0.3373
Atp1a3	ATPase, Na+/K+ transporting, alpha 3 polypeptide	0.3547
Slc5a1	solute carrier family 5 (sodium/glucose cotransporter), member 1	0.3701
Clca3	chloride channel calcium activated 3	0.3773
Kcnj15	potassium inwardly-rectifying channel, subfamily J, member 15	0.4207
Chi3l3 *	chitinase 3-like 3	0.4246
Slco4c1	solute carrier organic anion transporter family, member 4C1	0.4487
Man2b2	mannosidase 2, alpha B2	0.4520
***Immune/Defense Response***		
Ear11	eosinophil-associated, ribonuclease A family, member 11	0.2201
Chia *	chitinase, acidic	0.3222
Sod3	superoxide dismutase 3, extracellular	0.3353
Csf2ra	colony stimulating factor 2 receptor, alpha, low-affinity (granulocyte-macrophage)	0.3959
Ifitm2	interferon induced transmembrane protein 2	0.4031
Igf1	insulin-like growth factor 1	0.4424
*Osmr*	oncostatin M receptor	0.4612
***Cytokine/Chemokine***		
Ccl22	chemokine (C-C motif) ligand 22	0.2776
Tnfsf12	tumor necrosis factor (ligand) superfamily, member 12	0.3065
Ccl9	chemokine (C-C motif) ligand 9	0.4578
Il33 *	interleukin 33	0.4640
***Extracellular Matrix***		
Col6a2	collagen, type VI, alpha 2	0.3976
Muc5ac	mucin 5, subtypes A and C, tracheobronchial/gastric	0.4245
Muc4	mucin 4	0.4415
Col1a1	collagen, type I, alpha 1	0.4641
***Transcriptional Factor***		
Srebf2	sterol regulatory element binding factor 2	0.3294
Foxp4	forkhead box P4	0.3743
Nfic	nuclear factor I/C	0.4391
***MicroRNA***		
Mir5109	microRNA 5109	0.2007
Mir5107	microRNA 5107	0.4523
***Other***		
Syn2	synapsin II	0.0688
4833422F24Rik	RIKEN cDNA 4833422F24 gene	0.0775
LOC100048885	major urinary protein LOC100048885	0.1392
Corin	corin	0.1992
Zfp366	zinc finger protein 366	0.2274
Zfp385b	zinc finger protein 385B	0.2356
Epha7	Eph receptor A7	0.2597
Bahcc1	BAH domain and coiled-coil containing 1	0.2673
Actg2	actin, gamma 2, smooth muscle, enteric	0.2694
Phlda1	pleckstrin homology-like domain, family A, member 1	0.2729
Actc1	actin, alpha, cardiac muscle 1	0.2852
BC048546	cDNA sequence BC048546	0.2998
Trim65	tripartite motif-containing 65	0.3015
Bin2	bridging integrator 2	0.3016
Fcgbp	Fc fragment of IgG binding protein	0.3248
Prrc2a	proline-rich coiled-coil 2A	0.3288
Dusp8	dual specificity phosphatase 8	0.3328
Rhob	ras homolog gene family, member B	0.3346
Atn1	atrophin 1	0.3370
Rpph1	ribonuclease P RNA component H1	0.3560
Midn	midnolin	0.3735
Notch3	Notch gene homolog 3 (Drosophila)	0.3899
Sema5a	sema domain, seven thrombospondin repeats (type 1 and type 1-like), transmembrane domain (TM) and short cytoplasmic domain, (semaphorin) 5A	0.3949
Gm15401	predicted gene 15401	0.3967
Tgfb1i1	transforming growth factor beta 1 induced transcript 1	0.4005
Taok2	TAO kinase 2	0.4159
Samd4b	sterile alpha motif domain containing 4B	0.4693

Verified by RT-qPCR.

NCBI Gene Expression Omnibus accession numbers GSE49047 (http://www.ncbi.nlm.nih.gov/geo/query/acc.cgi?acc=GSE49047).

**Table 4:** Genes Upregulated in the Lungs of HDM-treated DDAH1-transgenic Mice versus HDM-treated Wild Type Mice.

Symbol	Name	Fold Change
***Ribosomal Complex***		
Rps21	ribosomal protein S21	5.4796
Rps12	ribosomal protein S12	5.4397
Rpl30	ribosomal protein L30	4.2587
Mrps16	mitochondrial ribosomal protein S16	4.1518
Rpl34	ribosomal protein L34	3.8427
Sec61b	Sec61 beta subunit	3.7764
Rps14	ribosomal protein S14	3.6956
Rpl36a	ribosomal protein L36A	3.6885
Rps15a	ribosomal protein S15A	3.6498
Rpl37a	ribosomal protein L37a	3.6295
Pttg1	pituitary tumor-transforming gene 1	3.5963
Rplp2	ribosomal protein, large P2	3.4879
Rps27	ribosomal protein S27	3.4532
Rpl27	ribosomal protein L27	3.4067
Rps28	ribosomal protein S28	3.3511
Mrpl20	mitochondrial ribosomal protein L20	3.2634
***Ribosomal Pseudogene***		
Gm11968	Rps15a pseudogene	4.4686
Gm12191	ribosomal protein L30 pseudogene	4.2620
Gm13253	ribosomal protein S15a pseudogene	4.0395
Rpl34-ps1	ribosomal protein L34, pseudogene 1	3.8391
Rplp2-ps1	ribosomal protein, large P2, pseudogene 1	3.3696
***Metabolism/Oxidative Process***		
Qdpr	quinoid dihydropteridine reductase	5.7248
Glrx5	glutaredoxin 5 homolog (S. cerevisiae)	5.0037
Ndufa2	NADH dehydrogenase (ubiquinone) 1 alpha subcomplex, 2	4.9044
Ndufs5	NADH dehydrogenase (ubiquinone) Fe-S protein 5	4.7132
Sumf1	sulfatase modifying factor 1	4.4089
Cox7a2l	cytochrome c oxidase subunit VIIa polypeptide 2-like	4.2862
Cox17	cytochrome c oxidase, subunit XVII assembly protein homolog (yeast)	4.1528
***Cytokine/Chemokine***		
Ccl20	chemokine (C-C motif) ligand 20	6.3854
Cxcl3	chemokine (C-X-C motif) ligand 3	3.9814
***Other***		
Dtwd1	DTW domain containing 1	14.2305
Flrt2	fibronectin leucine rich transmembrane protein 2	10.6123
1110038B12Rik	RIKEN cDNA 1110038B12 gene	8.9809
Scgb3a2	secretoglobin, family 3A, member 2	7.4584
Fpr2	formyl peptide receptor 2	6.5479
Fkbp14	FK506 binding protein 14	6.3078
Hbxip	hepatitis B virus x interacting protein	4.8871
BC002163	NADH dehydrogenase Fe-S protein 5 pseudogene	4.7683
S100a13	S100 calcium binding protein A13	4.6935
H3f3a	H3 histone, family 3A	4.6727
Commd1	COMM domain containing 1	4.6707
Osgep	O-sialoglycoprotein endopeptidase	4.2386
2010001M09Rik	RIKEN cDNA 2010001M09 gene	4.1526
Mettl7a2	methyltransferase like 7A2	4.1202
0610007C21Rik	RIKEN cDNA 0610007C21 gene	3.9575
Tmbim4	transmembrane BAX inhibitor motif containing 4	3.9312
Snrpe	small nuclear ribonucleoprotein E	3.9247
Plac9	placenta specific 9	3.7440
Gngt2	guanine nucleotide binding protein (G protein), gamma transducing activity polypeptide 2	3.5724
Tomm7	translocase of outer mitochondrial membrane 7 homolog (yeast)	3.5352
Gm9846	predicted gene 9846	3.4544
Ccdc84	coiled-coil domain containing 84	3.4251
S100a6	S100 calcium binding protein A6 (calcyclin)	3.3956
Vamp8	vesicle-associated membrane protein 8	3.3746
Shfm1	split hand/foot malformation (ectrodactyly) type 1	3.3414
Cd52	CD52 antigen	3.3025
Mettl7a1	methyltransferase like 7A1	3.2925
Pcolce2	procollagen C-endopeptidase enhancer 2	3.2669
Fam166a	family with sequence similarity 166, member A	3.2667
Neat1	nuclear paraspeckle assembly transcript 1 (non-protein coding)	3.2657

NCBI Gene Expression Omnibus accession numbers GSE49047 (http://www.ncbi.nlm.nih.gov/geo/query/acc.cgi?acc=GSE49047).

### The expression of IL-13, IL-4, IL-33, CCL11, ARG1, MMP-12, CHIA, CHI3L3 and CHI3L4 is decreased in the lungs from DDAH1-transgenic mice compared to wild type mice following allergen challenge

**Figure 6:**
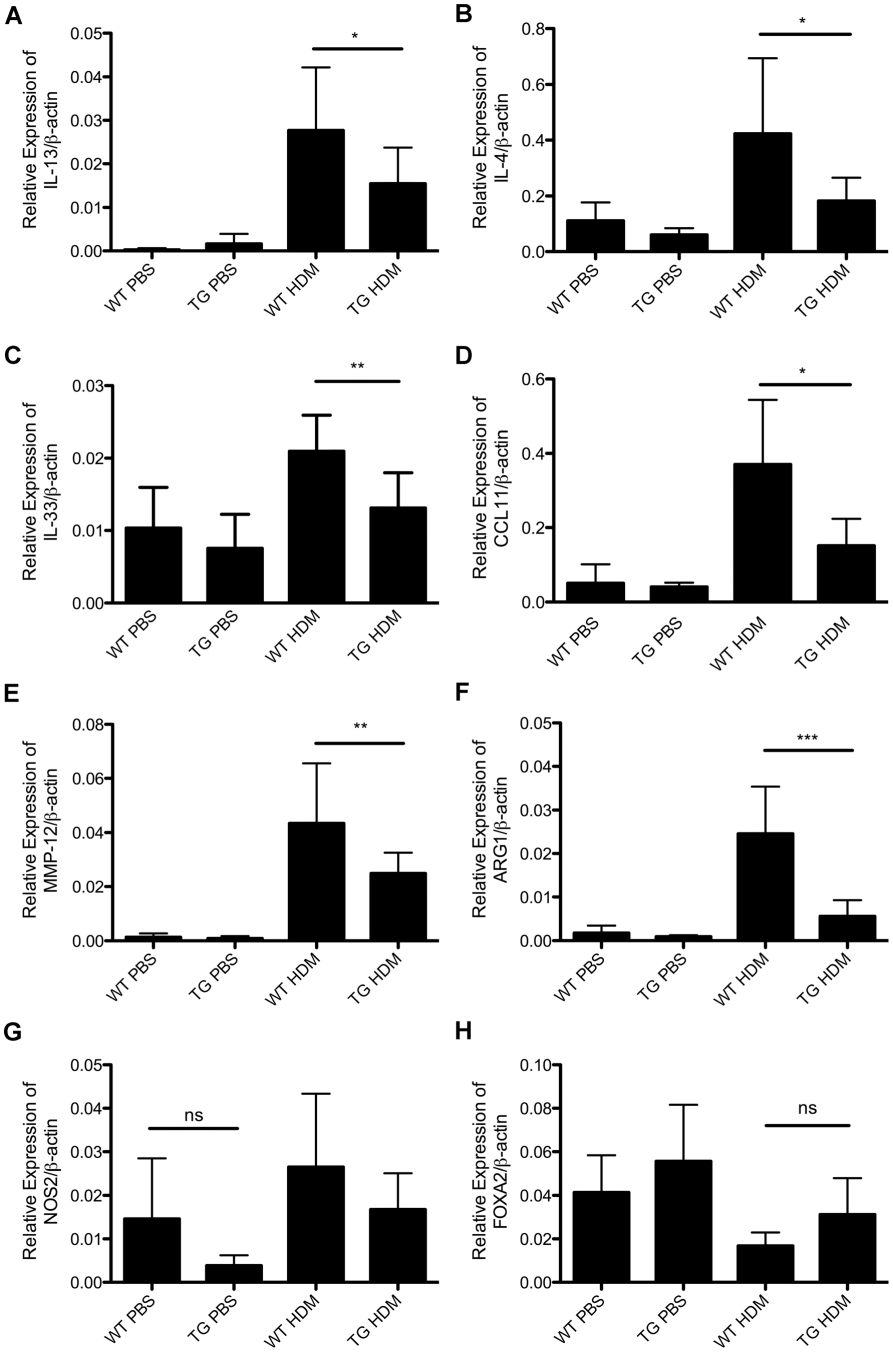
Expression of IL-13, IL-4, IL-33, CCL11, MMP-12, ARG1, NOS2 and FOXA2 in lungs from PBS or HDM-treated C57BL/6 wild type and DDAH1-transgenic mice. (A) IL-13. (B) IL-4. (C) IL-33. (D) CCL11 (E) MMP-12. (F) ARG1. (G) NOS2. (H) FOXA2. WT: wide type; TG: DDAH1-transgenic. Data are shown as mean±SD (n = 5-9). *, p<0.05; **, p<0.01; ***, p<0.001; ns, not significant.

**Figure 7:**
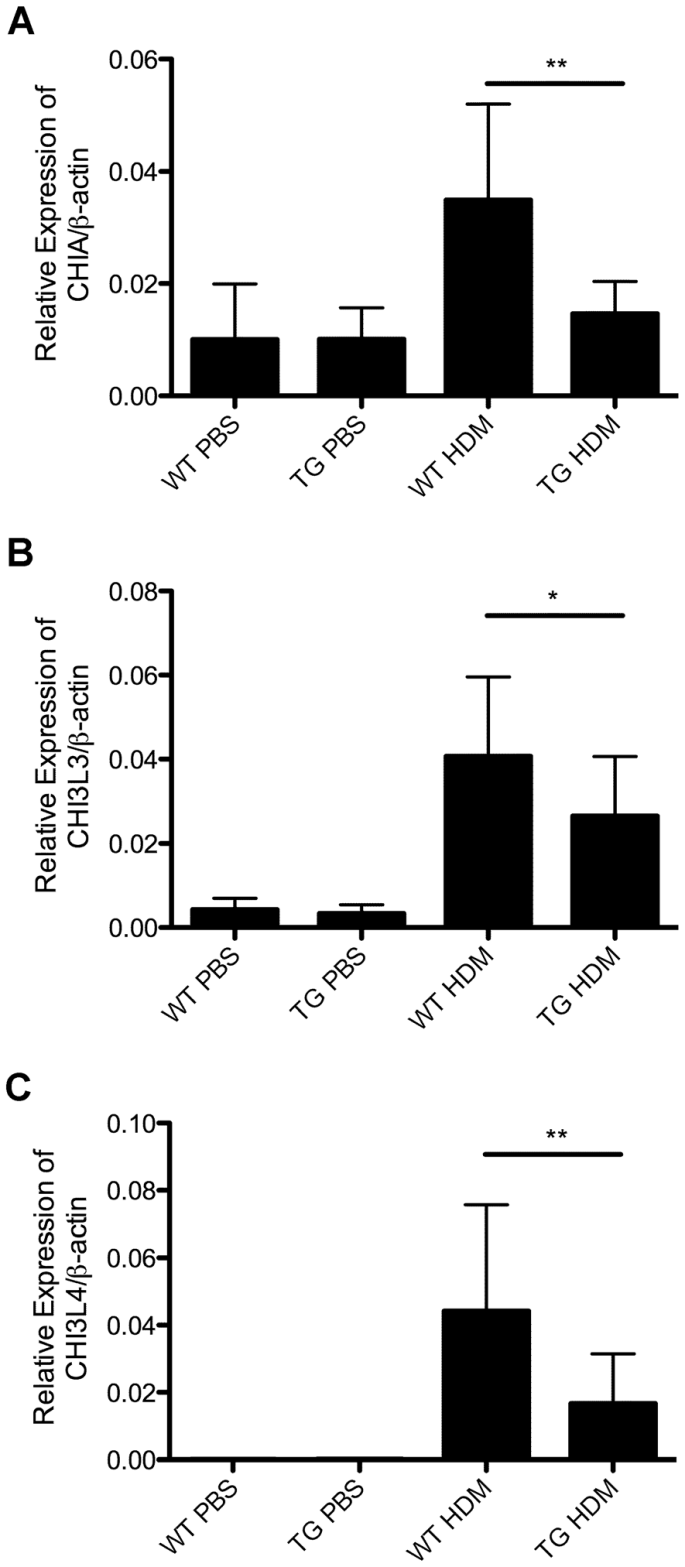
Expression of acidic chitinase (CHIA) and chitinase like proteins (CHI3L3 and CHI3L4) in the lungs of PBS or HDM-treated C57BL/6 wild type and DDAH1-transgenic mice. (A) CHIA. (B) CHI3L3. (C) CHI3L4. WT: wide type; TG: DDAH1-transgenic. Data are shown as mean±SD (n = 5–9). *, p<0.05; **, p<0.01.

Based on RNA-Seq results, we verified the expression of some key cytokines, chemokines, matrix metallopeptidases and chitinases including IL-13, IL-4, IL-33, CCL11, MMP-12 and CHIA. As shown in Fig. 6A and 6B, induction of IL-13 and IL-4 expression in the lungs was attenuated in HDM-treated DDAH1-transgenic mice compared to wild type mice. Expression of IL-33 was decreased in lungs from DDAH1-transgenic mice after HDM treatment compared to wild type mice (Fig. 6C). We found that the expression of CCL11 and MMP-12 was decreased in HDM-treated DDAH1-transgenic mice compared to HDM-treated wild type mice (Fig. 6D, 6E). The expression of ARG1, a key enzyme in arginine/NO metabolism, was significantly decreased in HDM-treated DDAH1-transgenic mice compared to HDM-treated wild type mice (Fig. 6F). The difference in expression of iNOS (NOS2) or FOXA2 was not significant between wild type and DDAH1-transgenic mice (Fig. 6G, 6H). The expression of acidic chitinase (CHIA) and chitinase like proteins (CHI3L3 and CHI3L4) was decreased in the lungs of HDM-treated DDAH1-transgenic mice compared to HDM-treated wild type mice (Fig. 7A–7C).

## Discussion

Our data reveal that expression of DDAH1 and DDAH2 is decreased in the lungs in a mouse model of asthma, and overexpression of DDAH1 attenuates allergen-induced airway inflammation. Asthma is a condition of decreased NO bioavailability. ADMA is an endogenous inhibitor of NOS, which is a major source of NO. DDAH1 and DDAH2 are responsible for metabolism of over 90% of ADMA *in vivo*, and our data support a role for DDAH downregulation in asthma pathogenesis.

The mechanism of the observed downregulation of DDAH may be epigenetic modulation. Studies using mouse trophoblast stem cells and trophoblastic tissues of postimplantation mouse embryos showed DNA methylation-dependent epigenetic regulation of DDAH2 gene expression. The CpG island in the DDAH2 promoter was hypermethylated in trophoblast stem cells but hypomethylated in differentiated cells [39]. We found that the mouse DDAH1 promoter also contains a CpG island [40], but their methylation status and relationship to gene expression are unknown. Previous studies have shown that allergen exposure results in altered methylation status of IL-4 and IFNγ promoter CpG islands [41]. It is likely that allergen exposure results in hypermethylation of mouse DDAH1 and DDAH2 promoter CpG islands, which results in decreased expression of DDAH1 and DDAH2 in mouse lungs.

Our data showed that overexpression of DDAH1 attenuated allergen-induced airway inflammation although it had no significant effect on AHR. It is not surprising as studies showed uncoupled airway inflammation and AHR in allergen challenged C57BL/6 mice due to strain-dependent genomic factors [42]. The infiltration of eosinophils into the lungs was decreased in HDM-exposed DDAH1-transgenic mice, which is consistent with the result that expression of CCL11 was decreased in lungs from HDM-treated DDAH1-transgenic mice. The total IgE and HDM-specific IgE levels in BALF or serum were decreased in DDAH1-transgenic mice after HDM exposure, suggesting that overexpression of DDAH1 affected Ig class switch.

The RNA-Seq data showed that overexpression of DDAH1 results in decreased lung expression of multiple immune/defense response genes that are associated with a network of inflammatory responses. The top upstream target is iNOS. A previous study showed that increased ADMA levels increase the expression of iNOS in mouse lungs [12], suggesting the effect of DDAH1 overexpression on iNOS expression/activity may be mediated by altered ADMA levels. Following HDM exposure, the expression of ARG1 is significantly decreased in the lungs from DDAH1 transgenic mice, suggesting arginine/NO pathways play important roles in the effect of DDAH1 overexpression on allergic airway inflammation. We found the expression of genes involved in mucus production (Clca3, Muc5ac and Muc4) and collagen synthesis (Col6a2 and Col1a1) is also decreased. Another previous study showed that increased levels of ADMA resulted in increased pulmonary collagen deposition [11], suggesting DDAH1 may regulate collagen synthesis through modulation of ADMA levels. Network analysis suggests that DDAH1 regulates mucus production gene expression through IL-13/FOXA2. RT-qPCR data further showed that the expression of IL-13, IL-4 and CCL11 is decreased in HDM-treated DDAH1-transgenic mice, which is consistent with attenuated eosinophil infiltration in airways and decreased serum and BALF IgE levels. Although the difference in expression of iNOS and FOXA2 is not significant between the wild type and DDAH1-transgenic mice, overexpression of DDAH1 may directly or indirectly affect the activity of iNOS and FOXA2. Interestingly, the expression of acidic chitinase (CHIA) and chitinase like proteins (CHI3L3 and CHI3L4) was decreased in the lungs of HDM-treated DDAH1-transgenic mice. Acidic chitinase has been shown to paly important roles in asthma [43]. CHI3L3 and CHI3L4 are rodent specific chitinase like proteins that are induced by Th2 cytokines or allergen challenge and have chemotactic activity [44]–[46]. Whether acidic chitinase and chitinase like proteins can modulate the expression of DDAH1 requires further studies. Overexpression of DDAH1 may have non-specific effects on gene expression. Generation of cell type specific transgenic mice with different expression levels of the DDAH1-transgene would help minimize the non-specific effects.

In summary, our data suggest that decreased expression of DDAH1 and DDAH2 in lungs may contribute to allergic asthma and overexpression of DDAH1 attenuates allergen-induced airway inflammation through modulation of Th2 responses.

## Supporting Information

Table S1 **Primers for PCR.**
(DOC)

Table S2 **Genes differentially expressed in the lungs of PBS-treated wild type and PBS-treated DDAH1-transgenic mice.** For RNA-Seq, equal amounts of RNA were pooled from each mouse lung in an experimental group (n = 4 per group) and analyzed in duplicate. The bioinformatics analyses were described in Materials and Methods. The differentially expressed genes with a p-value of <0.01 were shown. WT: wild type; TG: DDAH1-transgenic; Inf: infinity.
(XLS)

Table S3 **Genes differentially expressed in the lungs of HDM-treated wild type and HDM-treated DDAH1-transgenic mice.** For RNA-Seq, equal amounts of RNA were pooled from each mouse lung in an experimental group (n = 4 per group) and analyzed in duplicate. The bioinformatics analyses were described in Materials and Methods. The differentially expressed genes with a p-value of <0.01 were shown. WT: wild type; TG: DDAH1-transgenic; Inf: infinity.
(XLS)
