# Supplementary material for: Overexpression of Dimethylarginine Dimethylaminohydrolase 1 Attenuates Airway Inflammation in a Mouse Model of Asthma
Source: PLoS One. 2014 Jan 10;9(1):e85148. doi: 10.1371/journal.pone.0085148 (PMC3894860; doi:10.1371/journal.pone.0085148)
Supplement: Table S1 — Primers for PCR. (DOC) [file pone.0085148.s001.doc]

| **Primer** | **Sequence** |
| --- | --- |
| IL-13 forward | 5’-GCTTGCCTTGGTGGTCTCGCC-3’ |
| IL-13 reverse | 5’-GGGCTACACAGAACCCGCCA-3’ |
| IL-4 forward | 5’-CTGTAGGGCTTCCAAGGTGCTTCG-3’ |
| IL-4 reverse | 5’-CCATTTGCATGATGATGCTCTTTAGGC-3’ |
| IL-33 forward | 5’-TCCCAACAGAAGACCAAAG -3’ |
| IL-33 reverse | 5’- GATACTGCCAAGCAAGGAT-3’ |
| CCL11 forward | 5’-ATGAAAGGAGATGTGGGATTATT-3’ |
| CCL11 reverse | 5’-TTATCCTCAGTTACTCCTAACTCG-3’ |
| MMP-12 forward | 5’-TGGAGGTATGATGTGAGGCA-3’ |
| MMP-12 reverse | 5’-TTTGGTGACACGACGGA-3’ |
| ARG1 forward | 5’-CTCCAAGCCAAAGTCCTTAGAG-3’ |
| ARG1 reverse | 5’-AGGAGCTGTCATTAGGGACATC-3’ |
| NOS2 forward | 5’-GTTCTCAGCCCAACAATACAAGA-3’ |
| NOS2 reverse | 5’-GTGGACGGGTCGATGTCAC-3’ |
| FOXA2 forward | 5’-TCCGACTGGAGCAGCTACTAC-3’ |
| FOXA2 reverse | 5’-GCGCCCACATAGGATGACA-3’ |
| DDAH1 forward | 5’-GACGTCCTATTCACAGGCA-3’ |
| DDAH1 reverse | 5’-CAATCAGGTTGGGTCCG-3’ |
| DDAH2 forward | 5’-GGTTGATGGAGTGCGTAAA-3’ |
| DDAH2 reverse | 5’-GCTCCTCGATGATTGGTC-3’ |
| CHIA forward | 5’-CAAGCTACTTCTCGTCACAGG-3’ |
| CHIA reverse | 5’-GGCAGGGGTTAATGTCATCAG-3’ |
| CHI3L3 forward | 5’-CAGGTCTGGCAATTCTTCTGAA-3’ |
| CHI3L3 reverse | 5’-GTCTTGCTCATGTGTGTAAGTGA-3’ |
| CHI3L4 forward | 5’-TCCACTTTGAACCACATTCCAA-3’ |
| CHI3L4 reverse | 5’-CCAGCACTAACAGTAGGGTCA-3’ |
| β-actin forward | 5’-TCATCACTATTGGCAACGA-3’ |
| β-actin reverse | 5’-TGTGTTGGCATAGAGGT-3’ |

**Table S1. Primers for PCR**
